# The Role of Innate Leukocytes during Influenza Virus Infection

**DOI:** 10.1155/2019/8028725

**Published:** 2019-09-12

**Authors:** Prem P. Lamichhane, Amali E. Samarasinghe

**Affiliations:** ^1^Department of Pediatrics, University of Tennessee Health Science Center, Memphis, TN 38103, USA; ^2^Children's Foundation Research Institute, Memphis, TN 38103, USA

## Abstract

Influenza virus infection is a serious threat to humans and animals, with the potential to cause severe pneumonia and death. Annual vaccination strategies are a mainstay to prevent complications related to influenza. However, protection from the emerging subtypes of influenza A viruses (IAV) even in vaccinated individuals is challenging. Innate immune cells are the first cells to respond to IAV infection in the respiratory tract. Virus replication-induced production of cytokines from airway epithelium recruits innate immune cells to the site of infection. These leukocytes, namely, neutrophils, monocytes, macrophages, dendritic cells, eosinophils, natural killer cells, innate lymphoid cells, and *γδ* T cells, become activated in response to IAV, to contain the virus and protect the airway epithelium while triggering the adaptive arm of the immune system. This review addresses different anti-influenza virus schemes of innate immune cells and how these cells fine-tune the balance between immunoprotection and immunopathology during IAV infection. Detailed understanding on how these innate responders execute anti-influenza activity will help to identify novel therapeutic targets to halt IAV replication and associated immunopathology.

## 1. Introduction

Respiratory viruses infect millions around the world each year causing a range of symptoms and claiming thousands of lives [1–3]. Some viruses such as respiratory syncytial virus are lethal to the very young but harmless to most adults [4]. Other viruses, such as rhinovirus, cause essentially the same symptoms (predominantly a runny nose) in any age group [5]. Still others like influenza A virus (IAV) can cause severe infections in patients across age groups during one season, mild symptoms in another year or be lethal in yet another season [6]. In fact, IAV infections cause seasonal epidemics and global pandemics and are a major cause for public health concern. Although generally milder than pandemics, seasonal influenza epidemics can cause around 650,000 deaths globally each year [7]. Despite mainstay vaccination strategies to minimize IAV infections, influenza pandemics have occurred once every 10-30 years, primarily due to cross-species transmission and antigenic shifts in the virus [8, 9].

As a member of the Orthomyxoviridae family, IAV is an enveloped virus with an octasegmented negative sense, single-stranded RNA genome [6]. At present, 18 hemagglutinin (HA) and 11 neuraminidase (NA) subtypes of IAV are documented to circulate in nature [10]. Only two HA subtypes of IAV (H1N1 and H3N2) and two lineages of influenza B viruses (Victoria and Yamagata) cause annual epidemics in humans [6]. However, IAVs dominate, inducing more severe morbidity and mortality compared to influenza B viruses, therefore, will be the focus of this review [11–13].

Rapid changes in IAV surface antigens through antigenic shift have resulted in three pandemics during the 20^th^ century [14]. Avian IAV subtypes (H5N1, H7N1, H7N2, H7N3, H7N9, H9N2, and H10N8) can cross the species barrier to infect humans [8, 15, 16] and cause severe, lethal disease. In fact, H5N1 highly pathogenic avian influenza (HPAI) and the H7N9 low pathogenic avian influenza viruses pose a serious public health concern due to their respective high fatality rates of 52.79% and 39.42% [17]. The most severe H1N1 influenza pandemic occurred in 1918 claiming over 50 million lives [18] while the last H1N1 pandemic in 2009 (pH1N1) is estimated to have claimed ~200,000 lives across the globe [19]. While annual vaccinations are highly encouraged by public health agencies, poor adherence and low efficacy increase the need for better strategies to understand host responses during IAV pathogenesis in order to delineate other mechanisms which enhance antiviral immunity.

Primary and secondary immune barriers play a crucial role in safeguarding the host against influenza. Physical barriers of the immune system including soluble components like mucus, collectins, and antimicrobial peptides provide the first line of defense by mitigating virus exposure to underlying airway epithelial cells which are the principal site for IAV replication [20, 21]. Upon breaching these physical barriers, IAVs bind to sialic acid receptors on airway epithelial cells and enter these cells to complete replicative cycles within, destroying infected cells in the process [22, 23]. Predilections for specific sialic acid linkages may restrict IAV to the upper respiratory tract. However, alterations in the sialic acid linkage preferences, particularly in reassortant viruses, can render IAV more adept at lower respiratory tract infection and dissemination [24, 25]. Infection of the epithelium activates the innate branch of the immune system which consists of humoral/soluble as well as cellular components.

Infected airway epithelial cells trigger innate immune responses in two ways. First, viral RNAs within infected cells are sensed by pattern recognition receptors such as toll-like receptors, nucleotide-binding oligomerization domain-like receptors, or retinoic acid-inducible gene (RIG)-I like receptors. Signaling through these receptors induces the production of antiviral soluble factors, including interferons (IFNs), which act on adjacent healthy cells in a paracrine manner to trigger antiviral gene transcription. Activation of mediators like protein kinase R, 2′5′-oligoadenylate synthetase, RNaseL, cleavage and polyadenylation factor [26, 27] in otherwise healthy cells induces an antiviral state thereby limiting viral replication [28]. IFNs also induce expression of interferon-stimulated genes (like myxovirus-1) that have strong anti-influenza activity [29]. Furthermore, infected alveolar epithelial cells in the airway secrete proinflammatory cytokines like TNF*α*, IL-1*β*, IL-6, CCL2, CCL5, CXCL8, and CXCL10 [30] in superfluity, which support innate immune cell recruitment [20, 31] to contain viral spread. However, the production of inflammatory cytokines is not always protective as cytokine storms arise when they are secreted in excess as observed during HPAI H5N1 virus infection [32–34] often leading to severe pneumonia and death. Secondly, phagocytosis of infected cells by antigen-presenting cells (APCs) can activate adaptive immune responses [20, 28] that help to eliminate the infection.

Innate immune cells provide the first line of cellular defense to combat IAV infection. Leukocytes like neutrophils, monocytes, eosinophils, natural killer (NK) cells, innate lymphoid cells (ILCs), and *γδ* T cells provide anti-influenza host protection by releasing preformed cytokines and granule contents that either directly or indirectly help the host to eliminate the threat posed by replicating virus. On the other hand, macrophages and dendritic cells (DCs) can phagocytose IAV-infected cells and present viral antigens to T cells, initiating adaptive immune responses including B cell-mediated humoral immunity. The aim of this review is to provide insight into the function of these innate leukocytes in immune protection and repair of damaged airways upon IAV infection.

## 2. Neutrophils: The First Recruits

**Figure 1:**
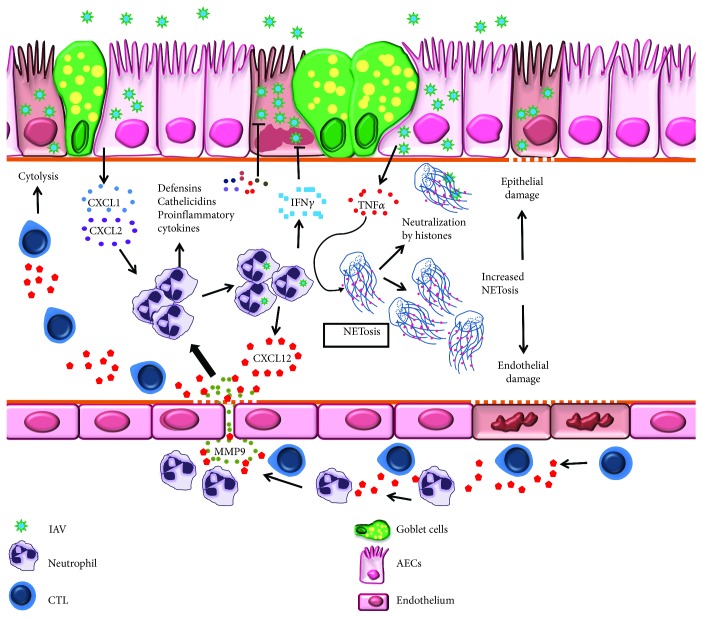
Schematic representation of neutrophil-mediated host defense mechanisms during influenza virus infection. Influenza A virus- (IAV)-infected airway epithelium releases neutrophil chemoattractants: CXCL1 and CXCL2. Neutrophils traffic into infected lungs by digesting endothelial basement membrane collagen. During trafficking, they release CXCL12-loaded vesicles/membranes which provide signals for CD8^+^ T cell migration and effector function. Once in the lungs, neutrophils secrete antimicrobial peptides and cytokines, including IFN*γ*, to inhibit IAV replication. TNF*α* produced by infected airway epithelium triggers the formation of neutrophil extracellular traps (NETs) which neutralize IAV particles. Enhanced NETosis damages airway epithelium and endothelium leading to severe pneumonia.

Neutrophils are among the first innate cells to be recruited during IAV infection. Neutrophils are granulocytes derived from hematopoietic stem cells influenced by IL-3, M-CSF, and G-CSF. While neutrophils are first to respond to any noxious stimuli, their role during influenza is complex and accumulation in the lungs is impacted both by virus strain and dose [35, 36]. Secretion of several chemotactic factors such as CCL3 [37], CXCL-1 (IL-8 in humans), and CXCL-2 [38] acts as chemotactic signals for neutrophil generation and recruitment. Neutrophils release MMP-9 to digest type IV collagen in the pulmonary endothelial basement membrane [37] and enter the lung tissue through CXCR2 engagement [39] (Figure 1).

Neutropenia has been demonstrated to increase the pulmonary virus titer and mortality rate upon IAV infection [40] suggesting a protective role for neutrophils during influenza. Phagocytosis, release of granular contents, and production of cytokines are major effector functions of neutrophils. These granulocytes contain cationic antimicrobial peptides like defensins and cathelicidins that neutralize IAV [41, 42]. Cathelicidins have anti-influenza activity *in vivo* through the reduction of viral replication and hindering the production of inflammatory mediators [41], showcasing a promising role for neutrophil antimicrobial peptides during virus infections. Furthermore, proinflammatory cytokines produced by neutrophils limit virus replication and halt progression to severe disease [43, 44] (Figure 1). Neutrophils are susceptible to IAV infection [45–47], although infection has been demonstrated to be both productive (for pH1N1) [48] and abortive (for seasonal H1N1 and WSN33) [45, 49]. These discrepancies might be related to virus subtypes/strains used. Interestingly, mice treated with *β*-defensin had reduced IAV burden and an associated reduction in neutrophils in the lungs [21], underscoring the importance of a prudently adjudicated immune response to host protection. Nonetheless, IAV-infected neutrophils upregulate IFNs and other antiviral factors [45, 46] that limit viral replication (Figure 1).

In addition to these conventional roles, neutrophils also utilize more sophisticated strategies to safeguard the host during influenza. One such scheme is the production of neutrophil extracellular traps (NETs) through NETosis, a release of web like structures of nucleic acids coated with histones [50] which capture viral particles [51, 52] thereby preventing viral dissemination. Inflammatory mediators like TNF*α* and cathelicidins in the pulmonary environment trigger NETosis [53, 54] (Figure 1). Arginine in histones within NETs has differential aggregation properties for IAV wherein seasonal strains are more likely to be inhibited compared to pH1N1 [55]. However, mechanisms that dictate strain preferences for NETosis in response to IAV are unknown. Increased NET production, as observed in severe cases of H7N9 and pH1N1 infection [56], augmented damage to the pulmonary endothelia and epithelia [56, 57] leading to severe pneumonia suggesting that uncontrolled NET production may contribute to disease severity.

Neutrophils were also shown to guide CD8^+^ T cell activation and recruitment into the lungs during influenza emphasizing the reliance of adaptive immune cells on those from the innate branch. By presenting IAV antigens on the cell surface, neutrophils function as APCs to activate CD8^+^ T cells within the lungs [58]. In addition, neutrophil membrane-bound CXCL12 is used as a beacon by CD8^+^ T cells during migration into the IAV-infected lungs [59] (Figure 1). The importance of neutrophils in innate immune defenses against IAV is well defined. Yet, uncontrolled trafficking of neutrophils as observed during infection with highly pathogenic H5N1 and 1918 pandemic IAV [44, 60] results in the production of excessive reactive oxygen species [61, 62] that causes oxidative stress-mediated pulmonary damage. Additionally, neutrophil CXCL12 trails can recruit CD8^+^ T cells [59] in excess which may boost/perpetuate inflammation, lung damage and severe pathology through cytolysis of infected epithelia during IAV infection. Thus, the abundance of neutrophils in the IAV-infected lungs may dictate their precise role in immunoprotection or immunopathology.

## 3. Monocytes: Recruited in Time of Need

Monocytes, peripheral blood phagocytes, are recruited into the lungs during IAV infection. Based on cell surface receptors, CD14 and CD16, circulating human monocytes can be subdivided into three distinct subsets: classical (CD14^++^/CD16^−^), intermediate (CD14^++^/CD16^+^), and nonclassical (CD14^+^/CD16^++^) [63–65]. Compared to peripheral circulation (where classical monocytes are predominant), intermediate monocytes predominate in airways [66]. Similarly, murine monocytes (CD11b^+^CD115^+^) are divided into three subsets based on the expression of Ly6C as classical (Ly6C^++^), intermediate (Ly6C^+^), and nonclassical (Ly6C^−^) [67, 68].

**Figure 2:**
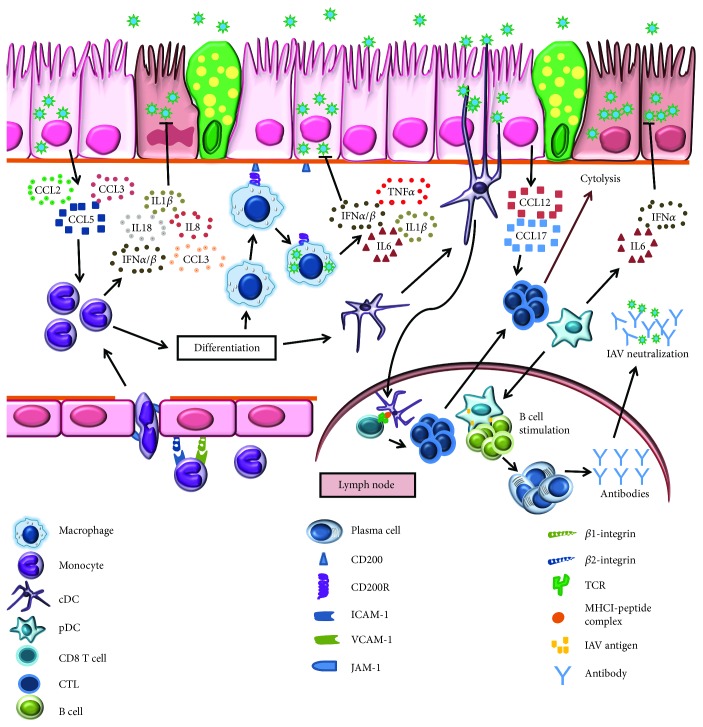
Schematic representation of monocyte-, macrophage-, and dendritic cell- (DC-) controlled immunoprotection and immunopathology during influenza virus infection. Influenza A virus- (IAV-) infected airway epithelium produces monocyte chemoattractants: CCL2, CCL3, and CCL5. Monocytes interact with endothelium through *β*1 integrin, VCAM-1, and *β*2 integrin, ICAM-1, binding, and subsequent entry into the lung tissue is assisted by JAM. These activated monocytes secrete inflammatory cytokines that inhibit IAV replication. Monocytes exert anti-IAV activity by differentiating into phagocytic cells like macrophages and DCs. IAV infection downregulates CD200-CD200R activating macrophages. These activated macrophages release an array of inflammatory cytokines that limit IAV replication. Both conventional and plasmacytoid DCs (cDCs and pDCs) exert anti-IAV activity in lungs. IAV-activated cDCs migrate to lymph node and present antigen to CD8^+^ T cells. Chemokines, CCL12 and CCL17, secreted by infected airway epithelium provide signals for the activated cytotoxic T cells trafficking into the lungs where they lyse IAV-infected cells. The other DC subset, pDC migrate to lymph node and stimulate B cell differentiation into antibody secreting plasma cells. These antibodies neutralize IAV particles conferring humoral protection.

The recruitment of these circulatory monocytes across the endothelial/epithelial barrier into the lungs has been extensively studied [69]. IAV-infected alveolar macrophages and epithelial cells secrete monocyte attracting chemokines, CCL2, CCL3, and CCL5 [69–71]. However, transepithelial migration of monocytes occurs through the CCL2/CCR2 axis, wherein CCR2-expressing monocytes move chemotactically towards CCL2 [69]. Interaction between monocyte integrins and endothelial adhesion molecules mediates transendothelial migration of monocytes [69, 72–75]. During IAV infection, monocyte transmigration across the endothelial barrier is predominantly dependent on interactions between VCAM-1 and *β*1 integrin (CD49d) and ICAM-1 interactions with *β*2 integrin (CD11a/CD18 or CD11b/CD18) [69] and promoted by TNF*α* secreted by infected alveolar macrophages (AMs) [69, 76], integrin-associated protein (CD47) [69, 76, 77], and junctional adhesion molecule 3 [69, 76, 78] (Figure 2).

Similar to neutrophils, monocytes are susceptible to IAV infection [79–81] irrespective of the subset [31], and infection rates may vary with the IAV subtype with the highest infectivity for H5N1 and H7N9 than pH1N1 [81]. Virus-infected monocytes secrete inflammatory cytokines and chemokines [31, 80] (Figure 2) in a virus dose-dependent manner [31]. Infected monocytes produce large amounts of IFNs [80, 82, 83] well known for their anti-influenza activity in addition to serving as a source for an array of cytokines and chemokines; for example, U937 promonocytic cells produce IL-1*β*, IL-8, IL-18, CCL3 [82], and IFN-*α*/*β* [80, 83]. Importantly, the microenvironment of infected lungs drives monocyte differentiation into macrophages and DCs (Figure 2) which play a pivotal role in phagocytosis of virus-infected cells thereby priming the adaptive immune system. During airway inflammation, migration and differentiation of blood monocytes contribute to the pool of pulmonary macrophages [20]. The absence of CD14 on monocytes/macrophages alters the immunopathogenesis during complex disease-disease interactions that occur when IAV infects hosts with underlying allergic states [84] suggesting that the function of each subset is regulated by the inflammatory milieu during influenza. The role of monocyte-derived macrophages in protection from IAV infection is described elsewhere [85, 86].

Although monocytes provide immunoprotection against IAV infection, excessive infiltration of monocytes can induce immunopathology and mice exhibiting uncontrolled IAV replication have excessive monocyte recruitment into the lungs [87]. Conversely, monocyte-deficient CCR2^−/−^ mice demonstrate increased survival following IAV infection [87–89], suggesting that monocytes can drive IAV-induced host pathology. Moreover, disease severity correlates with increased monocyte-derived cytokines [87] which in turn leads to immunopathology. Overall, the role in immune protection or pathology is determined by the number of infiltrating monocytes and the cytokine milieu.

## 4. Pulmonary Macrophages: Sentinels in Command?

The pulmonary macrophage population consists of alveolar macrophages (AMs) and interstitial macrophages (IMs) [86, 90]. Macrophages are phenotypically classified as proinflammatory (M1) and anti-inflammatory (M2). This classification is best suited for monocyte-derived macrophages wherein different cytokines and growth factors are used for differentiation but not AMs [86]. In relation to IAV, AMs are best studied while the role of IMs is ambiguous due to phenotypic similarity with monocytes and technical difficulty in liberating them from the lung tissue [91]. Irrespective of lower phagocytic ability compared to AMs [92], owing to their location in the interstitium, IMs phagocytose pathogens that have evaded AMs thereby providing a second line of defense within the tissue. The potential antiviral activity of IMs needs to be explored further.

As tissue resident professional phagocytes that safeguard the airway against intruding pulmonary pathogens, AMs maintain lung homeostasis [93, 94]. Cytokines, TGF-*β* and GM-CSF, promote differentiation of fetal monocytes into AMs [95–97]. With a prolonged half-life of about 1-5 weeks, the AM population is primarily maintained via self-renewal [98–100]. Pulmonary homeostasis is conserved in part by interaction of these AMs with alveolar epithelial cells (AECs). At steady state, AECs that express CD200 engage surface CD200R on AMs controlling the production of inflammatory cytokines [101]. However, IAV infection lowers the expression of CD200 and CD200R [101] compromising the CD200-CD200R axis which leads to macrophage activation and production of inflammatory mediators (Figure 2).

Pigs [102] and mice [103, 104] intranasally administered with clodronate liposome to deplete AMs have higher lung virus titers and severe disease characterized by impaired clearance of surfactant proteins, cellular debris, dead cells, pulmonary edema, and inflammation [102, 103]. Moreover, transgenic mice selectively depleted of AMs exhibit severe influenza [104, 105] suggesting a critical role for AMs in limiting virus-induced airway injury. However, the possible protective role of IMs may not be underestimated as they have more potent activators of antigen-specific T cells compared to AMs [90]. As most other leukocytes, AMs too secrete type I IFNs during IAV infection [106, 107] (Figure 2). IAV infection of AMs showed species specificity where human AMs are not infected while the murine counterparts exhibit abortive infection [108, 109]. Since IAVs fail to infect human AMs [108], an alternative mechanism like phagocytosis of apoptotic cells may also trigger AM activation and cytokine secretion during infection [110].

Owing to the contribution of AMs in maintaining lung tissue integrity and pulmonary homeostasis, their role in protecting AECs from IAV-induced damage is important. Type I AECs are responsible for gas exchange, and their impairment during infection leads to pulmonary dysfunction. On the other hand, type II AECs can self-renew or divide to replenish damaged type I AECs [111]. Engagement of CysLT, a cell surface cysteinyl leukotriene receptor on type I AEC, enhances their susceptibility to IAV infection [104]. However, AMs suppress the CysLT pathway in type I AEC [104] conferring the protection to AECs during infection. Peroxisome proliferator-activated receptor gamma (PPAR-*γ*), highly expressed in AMs and critical during their development [112], restricts AMs from excessive proinflammatory cytokine production [113] thereby maintaining lung homeostasis. Parallelly, PPAR-*γ* stimulation lessens IAV-associated inflammation within airways [114] and increases the secretion of tissue remodeling (MMP7 and MMP9) and epi-endothelial growth factors (EGF and VEGF) [115] suggesting a critical role for AMs as regulators of wound healing and tissue repair upon IAV infection.

The contribution of macrophages to immunopathology is evident from the fact that CCR2^−/−^ mice are protected from IAV-induced pulmonary tissue destruction and mortality [89]. Furthermore, protective mechanisms in these macrophage deficient mice are traced to delayed migration of T cells [89] suggesting that macrophages alter cellular immune responses. The role of macrophages in IAV-mediated pathology is based on findings from monocyte-derived macrophages (MDMs). The induction of macrophage-derived cytokines, IFN-*α*/*β*, TNF*α*, CCL2, CCL3, CCL5, and CXCL10, is greater for highly pathogenic H5N1 virus than seasonal influenza viruses [116, 117] suggesting that these inflammatory mediators may contribute to IAV-induced immunopathology. In contrast, MDMs infected with H5N1 virus showed lower expression of cytokines, namely, IFN-*α*/*β*, TNF*α*, CCL5, and CCL8 at early times postinfection [118]. This may be representative of an immune evasion strategy of IAVs to prolong uncontrolled replication and replication-induced pathology to gain a foothold in the host. Cumulatively, these observations suggest that pulmonary dysfunction observed in fatal cases of influenza depends on how macrophages respond to the invading virus.

## 5. Dendritic Cells: Calling for Help

Dendritic cells (DCs) are professional APCs that patrol the body surfaces (skin, gut, or airway) for intruding microbes or insults. They play a key role in host immunity by bridging innate and adaptive arms of the immune system [119, 120]. DCs are broadly classified as CD11c^+^ conventional DCs (cDCs) and CD11c^−^ plasmacytoid DCs (pDCs) [121, 122]. Furthermore, two subsets of cDCs have been identified in the airways: CD103^+^CD11b^−^ (CD103^+^ cDCs) and CD103^−^CD11b^+^ (CD11b^+^ cDCs) cells [123]; the latter of which resides in the lamina propria which lies immediately beneath the airway epithelium [124, 125]. In a steady state, the major DC population in the airway mucosa is CD103^+^ cDCs followed by CD11b^+^ cDCs and pDCs [124, 126, 127]. Humans also have more myeloid DCs (similar to cDCs in mice) in the airways than pDCs [66]. The CD103^+^ cDCs can extend their dendrites or traverse the alveolar epithelia into the airway for surveillance and antigen capture [123] (Figure 2).

Acute IAV infection leads to a decrease in cDCs and pDCs in peripheral circulation [128–130] and a sustained increase in the respiratory tract [129, 130] (Figure 2) suggesting active trafficking of DC populations during influenza. Following intranasal inoculation of IAV (X31 H3N2) in mice, pulmonary cDC subsets demonstrated sustained increase while it was transient for pDC. Moreover, draining lymph nodes (DLNs) contained only cDCs [127] suggesting maturation and migration of cDCs to DLNs following IAV infection (Figure 2). Antimicrobial peptide, *β*-defensin, induces a reduction in lung cDCs during pH1N1 infection [21], emphasizing the complex interaction and dependence between the soluble and cellular factors of the innate immune system. Depletion of CD103^+^ cDCs aggravated disease severity [127] suggesting a crucial role during influenza. Therefore, the totality of cDCs' contribution to lung health during IAV infection remains unknown, and since these subsets of cDCs secrete inflammatory mediators [131], a possible role in recruiting other leukocytes during influenza needs to be more fully explored.

Compared to other DC subsets, CD103^+^ cDCs are highly efficient at viral antigen uptake and migration to DLNs [131] and are the most efficient cross-presenters of the immune system [132] as they differ in their antigen processing and presentation capabilities [133]. While CD103^+^ cDCs process and present IAV antigens to CD8^+^ T cells efficiently [127, 131], IAV-activated CD11b^+^ cDCs fail to prime CD8^+^ T cells [127] (Figure 2). This might be related to the ability of these cells to support IAV replication *ex vivo*; CD103^+^ cDCs support productive IAV infection while CD11b^+^ cDCs do not [134], providing an explanation for CD103^+^ cDC's superior antigen-presenting capacity. However, whether CD103^+^ cDCs support IAV replication *in vivo* is unclear with data suggesting that viral antigen is acquired through phagocytosis instead to allow cross-presentation to CD8^+^ T cells [132].

The effective function of cDCs in priming the T cell-mediated immune response depends on (1) trafficking of IAV-activated DCs into DLNs, (2) presentation of antigen to specific CD8^+^ T cells, and (3) lung homing capacity of activated CD8^+^ T cells. Migration of pulmonary cDCs into DLNs depends on expression of CCR7, as *ccr7*-deficient mice lack DC trafficking into DLNs [133, 135]. Both CD103^+^ cDCs and CD11b^+^ cDCs express CCR7 [131] confirming the migratory nature of these cDCs. Once in the DLNs, these DCs prime IAV antigen-specific CD8^+^ T cells, which migrate into infected lungs in a CCR4-dependent manner [136] to exert anti-influenza activity (Figure 2).

Following IAV infection, pDCs produce large amounts of chemoattractants, importantly CXCL1, CCL2, CCL5, and CXCL10 [137] (Figure 2). Mice selectively depleted of pDCs exhibited delayed recruitment of T cells into airways [138] suggesting that they regulate accumulation of T cells during early IAV infection. The ability of pDCs to support IAV replication is dose dependent [139, 140]; susceptibility observed with higher multiplicity of infections (MOIs) [139, 140] might not correlate with infection in humans. Since, at lower MOIs, pDCs do not support IAV replication [139], they fail to prime CD4^+^ or CD8^+^ T cells directly [127]. However, pDCs can uptake IAV antigens from virus-infected cells and upregulate CCR7 expression in response [141] suggesting that they too can migrate to DLNs and cross-present acquired IAV antigens. During IAV infection, pDCs induce differentiation of B cells into antibody-secreting plasma cells through IFN*α* and IL-6 [142] and the depletion of pDCs abrogates antibody secretion [127]. The activation of lung-resident B cells during pH1N1 infection [143] may be led by these DC subsets that encounter viral antigen thereby providing dual protection at the site of infection as well as by couriering a call for help (Figure 2).

As with most factors of the immune response, moderation is required for health and excessive cytokine levels can be detrimental to the host. Highly pathogenic H5N1 virus-infected pDCs produce higher amounts of IFN*α* than those infected with less virulent seasonal H1N1 and H3N2 strains [139, 144]. While IFN*α* secreted from pDCs does have antiviral functions [139, 145] (Figure 2), prolific production during IAV infection results in uncontrolled inflammation and host pathology [146], alluding that pDCs may also contribute to the cytokine storm during infection. As an antithesis to sublethal IAV infection, lethal infection causes the accumulation of pDCs in DLNs with enhanced expression of Fas ligand (FasL) [147, 148] which engages with Fas, a membrane protein of the death receptor family [149] expressed on IAV-specific CD8^+^ T cells eliminating them via Fas-mediated apoptosis [147, 148]. Conversely, since IAV infection in pDC-depleted mice leads to enhanced accumulation of inflammatory cells, particularly CD11b^+^ cDCs and macrophages which produce massive amounts of proinflammatory cytokines (TNF*α* and IL-6) [150], pDCs may be important immunoregulators during lethal IAV infection. The protective versus deleterious effect of pDCs depends on the infectious dose.

## 6. Eosinophils: Additional Sources of Host Defense

**Figure 3:**
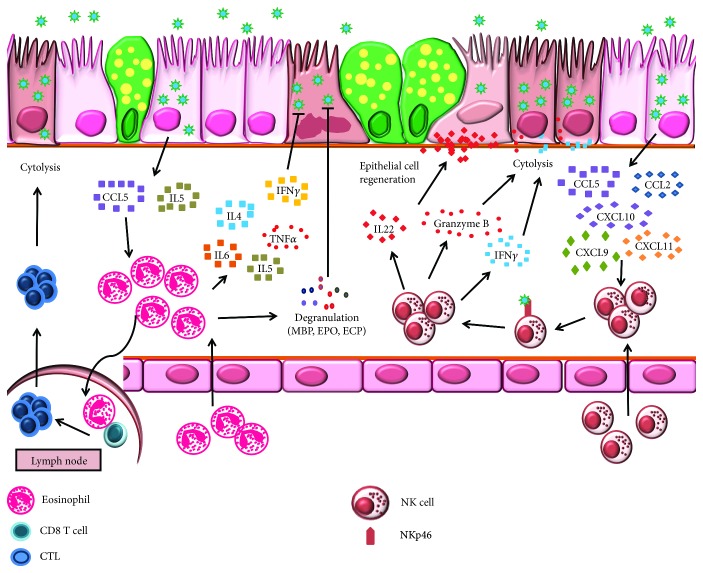
Immunoprotection and immunopathology mediated by eosinophils and natural killer (NK) cells during influenza. CCL5 and IL-5 released by IAV-infected airway epithelium mediate eosinophil trafficking into the lungs. Eosinophils undergo piecemeal degranulation and secrete inflammatory cytokines that exert anti-IAV activity. Additionally, IAV-infected eosinophils migrate to draining lymph nodes to present viral antigen to CD8^+^ T cells. Once activated, cytotoxic T cells traffic to the lungs to lyse IAV-infected cells. NK cell recruitment is driven by CCL2, CCL5, CXCL9, CXCL10, and CXCL11 secreted by IAV-infected airway epithelium. Engagement of IAV with NK cell receptor NKp46 activates NK cell effector functions. Perforins, granzyme B, and IFN*γ* released by NK cell destroy IAV-infected cells while IL-22 aids in the regeneration of damaged epithelium.

Eosinophils are not usually considered mediators of anti-influenza immunity. However, epidemiologic data associated with the 2009 H1N1 pandemic suggested that asthmatics, presumably with pulmonary eosinophilia, were less likely to suffer from IAV-induced morbidity and mortality [151–153]. Pediatric influenza patients who developed acute pneumonia demonstrated a rise in the serum IL-5 levels and peripheral eosinophilia [154], suggesting that eosinophil recruitment may be necessary for late-stage anti-influenza host defense. Pulmonary eosinophilia has also been documented in IAV-infected mice [155–158] suggesting that cytokines like CCL5 [30] or IL-5 [155] produced during IAV infection may drive eosinophil migration (Figure 3). Based on known functions of eosinophils during parasite infections and allergy [159], it may be likely that their influx into the lung during mid-late infection is in support of reparative processes required after IAV-induced cytopathology.

Eosinophils are conducive to IAV infection and undergo piecemeal degranulation in response to IAV [160]. This selective release of granule proteins such as major basic protein, eosinophil peroxidase, eosinophil cationic protein, and eosinophil-derived neurotoxin, in addition to immunoregulatory cytokines and chemokines [161, 162] at the infection site, may either immunomodulate other leukocytes or help contain the virus *in situ* (Figure 3). Additionally, mice suffering from acute allergy are protected from IAV-induced airway damage [157] suggesting that eosinophils are important mediators during anti-influenza immunity in special populations of patients such as those with a T_H_2 bias. Our findings that eosinophils were capable of trafficking to DLNs following IAV infection and their putative function in IAV antigen presentation in the context of MHCI to activate CD8^+^ T cells [160] forecast multifaceted functions for eosinophils during influenza pathogenesis [163].

## 7. Natural Killer Cells: On-Site Killing

As large granular lymphocytes representing about 10% of lung resident lymphocytes, NK cells accumulate in the respiratory tract in response to IAV infection [164–167]. This increase correlates with an initial decrease in circulatory NK cells [166, 168] suggesting that during early IAV infection NK cells are recruited directly from blood [164] (Figure 3). NK cell recruitment to the site of infection depends on expression of chemokine receptors, namely, CCR2, CCR5, and CXCR3 [164, 167, 169] through interaction with ligands like CCL2, CCL5, CXCL9, CXCL10, and CXCL11 [164, 167, 169] (Figure 3).

Primary effector functions (cytolysis of infected cells and cytokine secretion [170]) of IAV-stimulated NK cells are facilitated through cytotoxic receptors, NKp46, NKp44, and NKp30, of which viral HA binds directly to sialic acids expressed on NKp46 [171–173] (Figure 3). Viral subtypes may dictate the strength of NK cell activation wherein H5N1 and 1918 H1N1 viruses induce stronger responses compared to the pH1N1 virus [174], suggesting that the difference in receptor binding preference of the infecting virus differentially regulates NK cell activation. Cytolysis of IAV-infected cells is achieved by degranulation and release of perforin and granzyme, as well as by lytic activity of IFN*γ* secreted by activated NK cells [174] (Figure 3).

Epithelial cytopathology is a principal outcome of IAV infection of the respiratory tract which NK cells can mitigate through IL-22 production [165]. Airway epithelial cells expressing IL-22R [175] respond to IL-22 through epithelial regeneration which reduces inflammation [165]. This demonstrated the pivotal role of the IL-22-IL-22R axis in maintaining epithelial integrity and tissue homeostasis upon IAV infection (Figure 3). In vitro studies suggest that NK cell apoptosis triggered during IAV replication within these cells [171] may be an immune evasion strategy empoyed by IAV. Since the full spectrum of interaction between NK cells and IAV is yet to be elucidated, additional studies that focus on NK cells are warranted.

Cells that coexpress the NK cell marker and the T cell receptor, termed NKT cells, have gained more attention from influenza virologists in recent years. These cells provide immunoprotection during IAV infection by reducing inflammation, primarily the accumulation of inflammatory monocytes [176], reducing immunosuppressive activity of myeloid-derived suppressor cells [177], or through the production of IL-22 that controls lung epithelial damage [178].

## 8. Innate Lymphoid Cells: Immediate Depots of Cytokines

The ILC family consists of cytotoxic NK cells and three noncytotoxic members, ILC1, ILC2, and ILC3, that are innate counterparts of T cells that do not express antigen receptors [179]. Various insults activate ILC subsets; ILC1 responds to viruses and intracellular bacteria, ILC2 to extracellular parasites and allergens, and ILC3 to extracellular bacteria and fungi [179–182].

**Figure 4:**
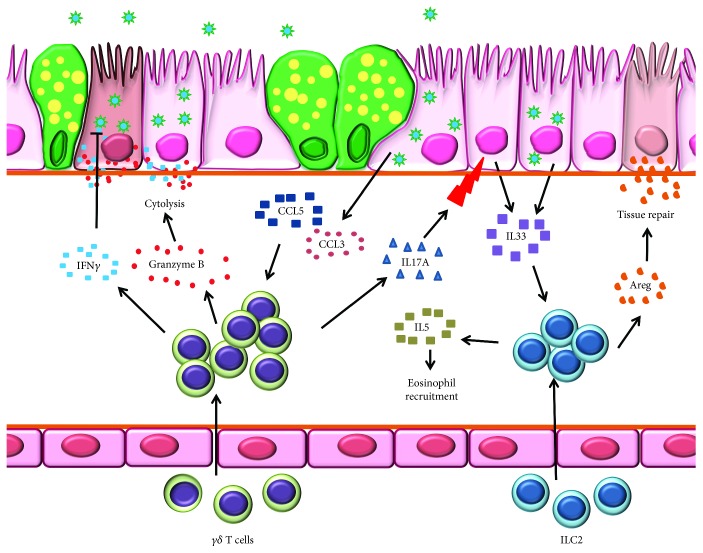
Innate lymphoid cells (ILCs) and *γδ* T cells are important during early stages of influenza virus infection. Schematic representation of immunoprotection and immunopathology mediated by ILCs in the IAV-infected lungs. *γδ* T cell migration into the infected lungs is driven along the CCR5-CCL5 axis. *γδ* T cells destroy infected cells through the secretion of IFN*γ* and perforin-granzyme B pathway and secrete IL-17A to trigger airway epithelium release of IL-33, a chemoattractant for ILC2. In addition, IL-33 secreted by IAV-infected epithelium enhances the trafficking of ILC2 into the lungs. Activated ILC2s release amphiregulin (Areg) which helps to repair tissue damage and IL-5 which induces eosinophil trafficking.

Although ILC2s are known to provide antihelminthic immunity, recent findings demonstrate their influx into lungs following IAV infection [183] most likely in response to alveolar epithelial- and macrophage-derived IL-33 [155, 184–187] sensed through surface-expressed IL-33R [188] (Figure 4). As potent producers of IL-5, ILC2s may regulate eosinophil infiltration during influenza [155, 188]. Within the lungs, ILC2s increase expression of genes encoding amphiregulin and extracellular matrix proteins asporin, decorin, and dermatopontin [188]. Strikingly, ILC2-derived amphiregulin is involved in tissue repair and remodeling [188] (Figure 4) suggesting that ILC2s are capable of lung tissue homeostasis during influenza. As major contributors of IFN*γ*, a crucial cytokine in the anti-influenza armamentarium, ILC1s induce cytolysis of IAV-infected cells [179–182]. However, IFN*γ* can also enhance influenza disease severity possibly through suppression of ILC2 function [189].

## 9. *γδ* T Cells: Holding Down the Fort

Innate-like T cells expressing *γ* and *δ* chains as receptors, *γδ* T cells constitute around 1-5% of blood lymphocytes. Given that *γδ* T cells respond antecedent to *αβ* T cells during infection [190], they may serve a pivotal role in early-stage antiviral host defense during influenza. Intravenous adoptive transfer of *γδ* T cells to IAV-infected mice inhibits viral replication and controls disease progression [191] suggesting that these cells can traffic to lungs (Figure 4). Moreover, IAV-activated *γδ* T cells express high levels of chemokine receptors CXCR5, CCR1, and CCR5 [192] allowing their migration towards CCL3 and CCL5 [193] abundant IAV-infected lungs [116] (Figure 4). As a means of self-promotion, IAV-activated *γδ* T cells produce preferred chemokines including CCL5 [192] to chaperon circulatory *γδ* T cell recruitment into the lungs. The production of CCL5 is dependent on virus subtypes (higher for avian viruses than human viruses [192]), indicating that infection with avian viruses promotes more robust *γδ* T cell infiltration.

Similar to other innate lymphocytes, *γδ* T cells exert anti-influenza activity either by direct killing [194, 195] or noncytolytic inhibition of virus replication through the secretion of IFN*γ* [192, 196] (Figure 4). The inhibition of human H1N1 virus replication is achieved by both noncytolytic and cytolytic mechanisms [192, 196]. In contrast, avian IAVs are resistant to the antiviral activity of IFN*γ*. The cytotoxicity of *γδ* T cells involves cytolytic granules (perforin and granzymes), NKG2D, TRAIL, and Fas-FasL pathways [194, 195]. Furthermore, *γδ* T cells effectively kill cells infected with various IAV subtypes [192, 195, 196] suggesting their role in the heterosubtypic immune response. It has been argued that IAV-infected macrophages and sentinel DCs alter the mevalonate pathway which in turn liberates isopentyl pyrophosphate (IPP), an antigen for *γδ* T cells [196]. The production of IPP activates *γδ* T cells, thereby conferring immune protection independent of incoming virus subtypes.

Subtypes of IAV differ in the degree of disease severity they elicit in the host. Apart from engaging the T cell receptor, IAV HA protein activates *γδ* T cells [197, 198] through *α*-2,3 and *α*-2,6 sialic acid receptor engagement on the cell surface [198] suggesting that HA-mediated differential activation of *γδ* T cells might be related to the degree of protection provided by these immune cells to IAVs with different sialic acid preferences.

The rapid secretion of IL-17A by *γδ* T cells from neonatal mice promotes AECs to produce IL-33 (Figure 4), protecting neonates from severe influenza [199]. Since *γδ* T cell-mediated IL-33 production serves as a cue for IL-33R expressing ILC recruitment [188], which in turn promotes tissue repair through amphiregulin secretion [199], the complex cell-cell interactions that occur even in the innate cell compartment of the immune system are necessary and important to anti-influenza host protection (Figure 4). These observations suggested that IL-17A and the timing of its release can modulate the balance between protection and pathogenicity in a host infected with IAV.

## 10. Conclusion

**Figure 5:**
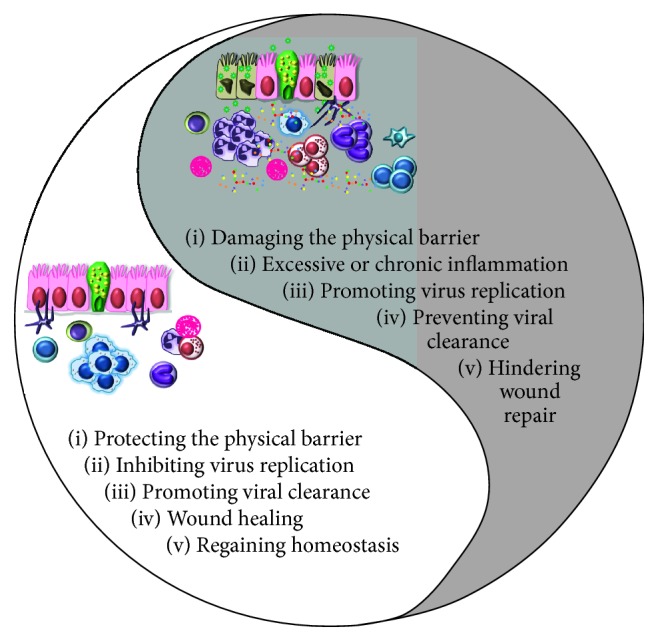
Yin and Yang of innate immune cell functions during influenza virus infection. At steady state, innate immune cells corporate to maintain pulmonary homeostasis while clearing IAV effectively with minimal tissue damage. When infected with a highly pathogenic virus or when immediate immune responses are unable to clear the infection, the immune system goes on overdrive causing excessive proinflammatory cytokine production and uncontrolled inflammation which can be pathogenic and detrimental to the host.

Despite annual vaccination strategies, IAV infections pose a continuous threat to human health. Development of better therapeutic options is required to tackle the growing burden of IAV infections. This requires a thorough understanding of virus pathogenesis and contribution of immediate responders during infection. Innate immune cells are critical to primary immunity against IAV infection at the respiratory barrier. However, the balance between innate immune cell-induced protection and pathology is governed by their abundance, the potency of secreted immune mediators, infecting viral strain/subtype, and immune status of the host (Figure 5). Hence, the identification of key regulators in innate leukocytes that mediate protection against IAV may provide broader options for therapeutic interventions.
